# Exploring the Role of Lactic Acid Bacteria Blends in Shaping the Volatile Composition of Fermented Dairy and Rice-Based Beverages: A Step towards Innovative Plant-Based Alternatives

**DOI:** 10.3390/foods13050664

**Published:** 2024-02-22

**Authors:** Iñaki Diez-Ozaeta, Laura Vázquez-Araújo, Olaia Estrada, Telmo Puente, John Regefalk

**Affiliations:** 1 BCC Innovation, Technology Center in Gastronomy, Basque Culinary Center, 20009 Donostia-San Sebastián, Spain; idiez@bculinary.com (I.D.-O.); oestrada@bculinary.com (O.E.); jregefalk@bculinry.com (J.R.); 2 Basque Culinary Center, Faculty of Gastronomic Sciences, Mondragon Unibertsitatea, 20009 Donostia-San Sebastián, Spain

**Keywords:** fermentation, sensory, aroma, Rate-All-That-Apply, lactic acid bacteria, SPME-GC–MS

## Abstract

Plant-based products are currently gaining consumers’ attention due mainly to the interest in reducing the consumption of foods of animal origin. A comparison of two fermentative processes utilizing dairy milk and a rice beverage was conducted in the present study, using a commercial lactic acid bacteria strain combination (CH) and a selected mixture of lactic acid bacteria from yogurt (LLV). Cell viability and physicochemical characteristics (total soluble solids, pH, total acidity) were determined to describe the samples before and after fermentation, as well as the volatile composition (gas chromatography–mass spectrometry) and the sensory profile (Rate-All-That-Apply test). Results of the analyses showed significant differences among samples, with a clear effect of the raw material on the volatile profile and the sensory characterization, as well as a significant effect of the microbial combination used to ferment the matrices. In general, the selected LLV strains showed a greater effect on both matrices than the commercial combination. Dairy samples were characterized by a volatile profile represented by different chemical families (ketones, lactones, acids, etc.), which contributed to the common descriptive attributes of milk and yogurt (e.g., dairy, cheese). In contrast, rice beverages were mainly characterized by the presence of aldehydes and alcohols (cereal, legume, nutty).

## 1. Introduction

Fermented milk products, such as yogurt, cheese, and kefir, have played a pivotal role in human nutrition and culture since ancient times. These foods are the result of milk fermentation by beneficial bacteria and yeasts. This process not only enhances longevity and safety by inhibiting spoilage and pathogenic microorganisms but also improves nutritional value and digestibility [1,2]. Throughout the world, around 400 generic names are applied to traditional and industrialized fermented milk products, underscoring their deep entwinement with regional culinary traditions. Despite the diversity in nomenclature, these products often share core characteristics, leading to a more concise classification based on the type of milk used, the dominant microbial flora, and their principal metabolites. Such classifications include lactic fermentations, yeast–lactic fermentations, and mold–lactic fermentations [2].

The art of fermenting dairy milk has been honed over centuries, with the role of lactic acid bacteria (LAB) in this process thoroughly characterized by the scientific community. However, as the world pivots towards plant-based diets, the intricate molecular characteristics of fermenting plant-based beverages remain little explored. Current trends indicate a shift in consumer preference towards plant-based dairy alternatives. This shift is partly attributed to considerations of dairy farming’s environmental impact, ethical concerns, and health issues such as lactose intolerance [3]. While plant-based options are becoming more popular, they still face challenges in mimicking the sensory and textural qualities of traditional dairy products. The global dairy alternatives market, valued at USD 26.01 billion in 2022, is projected to grow at a robust CAGR of 12.6% from 2023 to 2030. This growth is propelled by evolving consumer preferences, with an increasing demand for plant-based options such as soy, almond, and rice beverages. The market is also witnessing a surge in demand for nutritional dairy alternatives, driven by consumers seeking low-calorie, high-protein, and vitamin-rich options. This shift towards dairy alternatives is not only a result of changing diets and eating patterns but also reflects a growing interest in sustainable and ethical food choices [4].

Fermented milk products are esteemed not only for their nutritional richness but also for their distinctive sensory properties, of which aroma plays a crucial role in consumer acceptability. This attribute is predominantly determined by a variety of volatile compounds, often at low concentrations, which are the result of intricate biochemical transformations of carbohydrates, lipids, and proteins conducted by the action of starter bacteria. For example, fermented milk has been suggested to contain over 100 different volatile compounds, with specific cultures producing signature flavors, such as acetaldehyde in yogurt or diacetyl in butter and buttermilk [5,6]. The volatile composition of such products is influenced by multiple factors, including the milk source, fermentation by specific bacterial species, and the subsequent processing and storage conditions. The volatile aroma compounds of dairy yogurts have been studied extensively during the last decades through gas chromatography–mass spectrometry (GC–MS). In contrast, research on plant-based fermented dairy alternatives has been more limited, primarily focusing on texture and potential probiotic properties [7,8,9,10]. A comparative study has shown that ketones were the main volatile flavor compounds across dairy, soy, and oat-based yogurts. The volatile profiles of dairy and oat-based yogurts were relatively similar, whereas soy-based yogurt was distinctly different, with high odor activity values of hexanal, 1-octen-3-one, 1-octen-3-ol, and 2-octenal [11]. Rice beverages fermented using different LAB strains showed 47 different volatile compounds, including acids, aldehydes, esters, furan derivatives, ketones, alcohols, benzene derivatives, hydrocarbons, and terpenes [12]. This research highlighted significant differences in the volatile profiles of the fermented beverages depending on the LAB starter (obligatorily homofermentative/facultatively heterofermentative and obligatorily heterofermentative), underscoring the influence of bacterial metabolism on the flavor and aroma of fermented foods.

The relationship between some sensory descriptors and the presence of specific volatile compounds in plant-based beverages has been previously studied [13,14]. Grainy, grassy, or beany off-flavors have been previously associated with plant-based beverages made from soy, oats, and peas [13]. Cereal/pseudocereal-based (e.g., oat, rice, quinoa) and nut-based beverages (e.g., almond, hazelnut, cashew, Brazil nut) were described as having a cereal taste and odor, and a nutty taste and odor notes, respectively [14], and could be the best contenders to serve as a base for these fermented products due to their neutral flavor profiles [14,15]. While the presence of volatiles in these raw materials has been explored, research on their transformation through LAB fermentation remains to be conducted. The sensory neutrality of some plant-based beverages provides an ideal canvas for the complex but subtle aromatic profiles produced by fermentation. The present paper delves into the transformative potential of microorganisms in refining the flavor of fermented plant-based beverages. An analysis of the performance of two different bacteria blends was carried out. These microbial combinations were used to ferment both dairy milk and a rice beverage under controlled conditions. Their acidification performance, cell viability, and effect on volatile profile modification were evaluated. In addition, a Rate-All-That-Apply test was conducted using a chefs panel to determine the sensory differences among samples.

## 2. Materials and Methods

### 2.1. Bacterial Strains and Growth Conditions

This study compared two starter culture combinations, each comprising two strains of LAB. The first combination, referred to as CH, was the commercial starter culture Vega^TM^Premium (Chr. Hansen A/S Hørsholm, Denmark) formed by conventional yogurt strains *Lactobacillus delbrueckii* spp. *bulgaricus* and *Streptococcus thermophilus*. The second combination, referred to as LLV, was formed by the strains *Lactococcus lactis* subsp. *lactis bv. diacetylactis* ViP1 and *Leuconostoc mesenteroides* subsp. *cremoris* ViEPS1, both of which were isolated from viili-like yogurt. In this latter case, bacteria were grown at 30 °C and propagated in liquid MRS (Man, Rogosa and Sharpe medium, Sigma Co., Darmstadt, Germany) supplemented with 2% glucose (MRSG).

### 2.2. Dairy and Rice Beverage Fermentations

Both combinations were inoculated in dairy milk (Gurelesa, Gipuzkoa, Spain) and a rice beverage (Yosoy, Girona, Spain). The nutritional composition of both beverages is shown in Table 1. Fermentations were carried out in 500 mL, and analyses were performed in triplicate. Each replicate was made from a different commercial lot of each beverage. These were also analyzed as control samples (without inoculation) to understand the impact of fermentation. CH bacteria blend was inoculated following supplier instructions: 0.02% of the thawed starter culture was directly inoculated into the beverages and incubated for 9 h at 43 °C (ICN 120 plus incubator, ARGO LAB, Barcelona, Spain). For LLV combination, overnight independent cultures of LAB strains grown in MRS with 2% glucose underwent centrifugation at 8000× *g* for 5 min at 20 °C (MicroStar12 centrifuge, VWR, Barcelona, Spain), followed by a wash with PBS 1X (PanReac, Barcelona, Spain) and a subsequent centrifugation under identical conditions. Then, cultures were resuspended in the corresponding beverage to an initial cell density of approximately 5 × 10^7^ CFU/mL. Fermentations performed by LLV were carried out for 9 h at 30 °C. Samples were collected at 0 h and 9 h for subsequent analytical determinations. After 9 h of fermentation, samples were stored at 4 °C.

### 2.3. Evaluation of Cell Viability

Samples were evaluated at inoculation time (0 h) and after fermentation (9 h). Serial decimal dilutions were carried out in PBS 1X (PanReac AppliChem, Barcelona, Spain) to determine the lactic acid bacteria count [11]. Bacterial enumerations were performed by taking 1 mL of each beverage sample, diluting with 9 mL PBS 1X, and making considered serial dilutions. Then, 100 µL of the corresponding dilution was spread on MRS agar, and plates were incubated for 48 h at 30 °C for the LLV combination and at 43 °C for the CH combination.

### 2.4. Determination of pH, Total Soluble Solids, and Total Acidity

The pH was determined at 20 ± 0.5 °C using a digital pH meter (Crison Basic 20, Hospitalet de Llobregat, Spain) calibrated with standard buffer solutions. Total soluble solids (TSS) were determined by refractometric method using a manual hand refractometer (VWR Inc., Darmstadt, Germany) with the results expressed in °Brix. The total acidity (TA) of samples was analyzed using a titration method with a standard solution of sodium hydroxide (0.1 N). 10 mL of each sample was diluted with 40 mL of distilled water and titrated with 0.1 mol/L of NaOH solution to a target pH 8.10 ± 0.02. Results were expressed as g of acetic acid per liter of sample. All determinations were conducted in duplicate.

### 2.5. Analysis of Volatile Composition

The volatile composition of the samples was determined by headspace solid-phase microextraction (HS-SPME) in combination with gas chromatography coupled with mass spectrometry using the same conditions as previously reported [16]. A total of 6 g of sample was weighed, and NaCl (0.6 g) was added into a 40 mL vial with polypropylene caps and PTFE/silicone septa. A 50/30 mm DVB/CAR/PDMS (Divinylbenzene/Carboxen/Polydimethylsiloxane) fiber (Supelco by Sigma- Aldrich Corp., St. Louis, MO, USA) was exposed to the sample headspace for 50 min at 40 °C and stirred at 250 rpm in the automatic injection port AOC 6000 Plus Auto Sampler (Shimadzu Scientific Instruments, Inc., Columbia, MD, USA). The separation and identification of compounds was conducted by gas chromatography, using a GC2030 (Shimadzu Scientific Instruments, Inc., Columbia, MD, USA), with a Sapiens X5MS column (Teknokroma, Barcelona, Spain; 30 m, 0.25 mm i.d., 0.25 μm film thickness), and coupled with a mass spectrometer detector (TQ8040 NX triple quadrupole mass spectrometer; Shimadzu Scientific Instruments, Inc., Columbia, MD, USA). The volatile compounds were identified using 3 methods: (a) GC–MS retention time of the chemical pure compounds, (b) retention indexes (RI) calculated using a commercial alkane standard mixture (Sigma-Aldrich, Steinheim, Germany), and (c) comparison of the compound mass spectrum with those of databases (NIST, 2024) [17]. Concentrations of aroma compounds were calculated by the application of the internal standard method [18] and expressed as mg/kg resulting from the relative peak area to the internal standard (2-methyl-3-heptanone, 0.33 mg/kg; Sigma-Aldrich, Steinheim, Germany).

### 2.6. Sensory Analysis

To determine the sensory properties altered by the fermentative process, as well as the main differences among products, a Rate-All-That-Apply (RATA) test was chosen. RATA is a sensory method that allows participants to select relevant sensory attributes for a sample from a list and then rate the intensity on a scale [19]. A 5-point scale was used in the present study, with 1 = “very low intensity” and 5 = “very high intensity”. The flavor attributes list was gathered from literature, including descriptors from fermented milks and plant-based beverages [14,20]. The list included the terms sweet, sour, bitter, salty, umami, cereal, legume, nutty, earthy, dairy, fruity, cheese-like, hay, toasted, metallic, soapy, oily, astringent, watery, graininess, thickness, and lumpiness. A panel of 10 assessors, chefs trained for the Rate-All-That-Apply test and with wide knowledge of sensory attributes identification and discrimination, participated in the study. The session was conducted in a tasting room with controlled temperature and relative humidity (21 ± 2 °C; 55 ± 5% RH); the illumination was a combination of natural and artificial light (fluorescent). Samples (approximately 20 mL) were randomly served at 6–8 °C in 40 mL transparent disposable cups, coded with 3-digit numbers. Water was provided for palate cleansing between samples.

### 2.7. Statistical Analysis

All values are expressed as the mean ± standard deviation (SD) of the three batches. Data from the fermented beverages was analyzed by two-way analysis of variance (ANOVA) using “type of beverage” and “type of inoculum” as factors, followed by Tukey’s (HDS) post hoc test (*p* value < 0.05). A partial least square regression (PLS regression map), with the intent of correlating volatile composition with the aroma sensory attributes that were significantly different among samples, was also conducted. All statistical analyses were performed using XLSTAT Version 2023.3.0 (Addinsoft, Paris, France).

## 3. Results and Discussion

### 3.1. Microbiological and Physicochemical Characterization of Fermented Beverages

Table 2 shows the results of the physicochemical and microbiological analyses of the fermented beverages. Significant differences were found among samples by type of beverage and type of inoculum in all studied parameters (pH, TA, and TSS), suggesting a different effect of the inoculated microorganisms on the two studied matrices.

**Figure 1:**
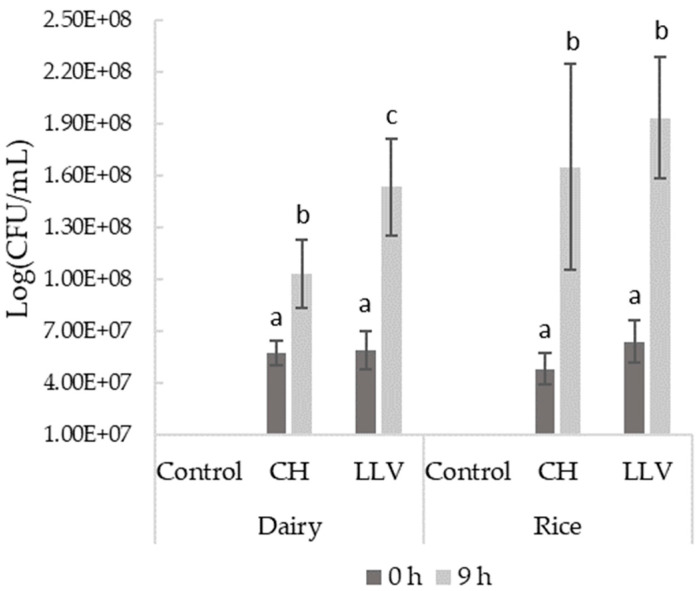
Results of microbiological analyses in the control and fermented samples. Different letters within the rows indicate different post hoc groupings by Tukey’s HSD (*p* < 0.05).

The correct development of the chosen bacterial combinations was observed during fermentation, with both microbial combinations being able to double bacterial density (Figure 1). Initial microbial populations were comparable in the two matrices at the time of inoculation, with concentrations ranging from 5–7 × 10^7^ CFU/mL. After inoculation, cell viability grew significantly to the range of 1–2 × 10^8^ CFU/mL in all samples. LLV combination presented a significantly greater growth in both the rice and dairy-fermented beverages compared to the commercial starter (CH). However, differences were only significant in the dairy milk samples. Direct inoculation of cultures, as recommended by the manufacturer, maybe a disadvantage compared to the preparation of fresh cultures ready for inoculation [21]. The matrix seemed to influence the correct development of both starter combinations, with higher bacteria counts in the rice-based beverages. A lower complexity of the matrix and a higher proportion of carbohydrates may partly account for the greater development of cultures in the rice drink.

The standards of the *Codex Alimentarius* state that dairy milk yogurt should contain a minimum of 7 log CFU/mL as the total of lactic acid bacteria microorganisms constituting the starter culture [22]. All the fermented beverages in the present study met this requirement. Dairy products have been widely studied as probiotic carriers, but the presence of live cultures in plant-based beverages is yet to be consciously measured to compare the potential benefits of these plant-based products with fermented dairy beverages. The results of the present study corroborate previous research in which plant-based matrices supported probiotic growth and maintained satisfactory viability throughout the fermentation process [23,24,25]. In addition, the correct application of cultures and high bacterial counts may play an important bioprotective role, reducing microbial contamination, improving product stability, and shortening fermentation times, all of them beneficial outcomes from an industrial perspective [26].

In addition to microbial viability, dairy and fermented milk products must meet certain quality requirements, such as specific pHs and TAs, the main indicators of proper fermentation processes [15]. Carbohydrate metabolism is the primary driver of product acidification, with lactic acid production being the main contributor to the observed pH decline during fermentation, imparting the distinctive sour flavor commonly associated with yogurt and other fermented beverages [10]. The fermentative capability and pH-lowering ability of all bacterial blends were confirmed during the present study (Table 2), and the results observed in the bacterial counts perfectly correlated with the measured pH values. A greater reduction of pH was observed in the samples fermented by LLV, and the fermented rice beverage had a lower pH than the fermented dairy milk. The results of the statistical analysis showed that the bacteria blend had a greater influence on the pH decline than the matrix, with the LLV combination showing superior performance.

Although many countries lack specific regulations that indicate the characteristics that a yogurt or a fermented dairy product must have, Spanish legislation establishes a pH < 4.6 for this food category [27]. The dairy milk sample fermented using LLV met this criterion, whereas the sample fermented using CH culture did not, potentially due to the fermentation time established in the present research. The fermentation time was a limiting factor in this sense, but it elucidated the different fermentative capacities of both microbial combinations. On the other hand, the selected strains showed considerable potential for developing plant-based fermented derivatives by effectively reducing pH to meet safety and quality benchmarks in a short time. This fermentation process emerges as a promising approach for formulating clean-label plant-based beverages, eliminating the need for artificial acidity regulators typically employed to adjust the pH of dairy products [3].

A significant increase in TA was observed in all samples due to fermentation, although significant differences were found among samples (Table 2). Statistical analysis suggested that the matrix had a greater influence than the bacterial blend on TA. In line with pH and CFU/mL results, LLV fermented samples showed higher TA, confirming their better performance over the CH bacterial blend. All samples showed a negative correlation between pH and TA, which is consistent with the behavior of fermented foodstuffs [28]. A higher concentration of acids after fermentation, mainly organic acids such as lactic, acetic, formic, etc., leads to a higher concentration of hydrogen ions, which reduces the pH. However, other intrinsic compounds of the food matrix could play a relevant role. The presence of higher levels of proteins, lipids, and citrate in dairy milk compared to rice matrix might contribute to the lower TA observed in fermented rice beverages, regardless of the bacterial blend employed for fermentation [29,30]. Bacterial degradation of proteins into amino acids, degradation of lipids into free fatty acids (e.g., butyric acid), and the metabolism of citrate towards the production of pyruvic and lactic acid contribute to a further increase in TA in fermented dairy beverages [31]. The *Codex Alimentarius* indicates a minimum TA of 0.6 g lactic acid/100 mL for yogurt-like products [22]. Therefore, the fermented dairy milk samples from the present study met this criterion, with values of approximately 0.6–0.7 g lactic acid/100 mL, while fermented rice beverages did not, having a TA of 0.03–0.09 g lactic acid/100 mL. These low values were in line with the results of previous research, which characterized new fermented plant-based beverages [32,33].

TSS results also underscored the significance of the original matrix in the resulting fermented beverage. Reduction of TSS was only significantly different in the fermented dairy beverages (Table 2). Rice-based beverages did not show such a reduction in TSS, probably due to the carbohydrate profile of the rice matrix. TSS is directly linked to the concentration of lactose, glucose, and galactose in dairy milk, while the carbohydrate profile is more complex in rice beverages [34]. The major carbohydrate of rice is starch (up to 80%), although it contains some free sugars such as glucose, sucrose, and dextrin. Therefore, the minor impact on reducing total solids could be ascribed to the considerable proportion of starch in the matrix. Additionally, the biological acidification seen in the fermentation of plant-based matrices not only contributes to a decrease in the starch hydrolysis index but also promotes the formation of resistant starch, which can ultimately lower the glycemic index, improving the digestibility and the healthy status of the final product [35].

### 3.2. Volatile Composition of Fermented Beverages and Its Relationship with Their Sensory Properties

Significant differences were detected among the fermented beverages’ flavors, as shown in Table 3. RATA results showed that the main differences among samples were due to the beverage type, with the rice beverage and its derivatives exhibiting more cereal, legume, nutty-like, and watery characteristics than the dairy products. The rice beverage used in the present study was mainly characterized by being sweet, watery, and having cereal aroma notes. Similar flavors were reported in a previous study in which up to 90 different plant-based beverages had been characterized using a RATA sensory test and GC–MS-olfactometry [14]. In general, plant-based beverages have been reported to have different kinds of off-flavors, such as beany and painty aromas or a chalky mouthfeel [36]. However, the sensory profile is quite variable, even within a specific product category [14]. Results of the present research confirmed the presence of some legume and nutty aromas in the rice beverage, also extending to the fermented samples, suggesting that the fermentation process did not diminish the intensity of these flavor notes. Although previous research reported some non-desirable flavors, such as hay-like or astringency, in different rice beverage brands, the sample used in the present study was not characterized by these additional notes.

The quality guidelines for USDA specifications report that the flavor profile of yogurt should be “acid, free from undesirable flavors such as bitter, rancid, oxidized, stale, yeasty and unclean” [20]. In this study, fermentation reduced sweetness and wateriness and increased sourness, cheese-like, and thickness in dairy milk, but it had no such effect on the rice beverage. Although some flavor trends could be observed in the rice beverages results, the only significant difference was that the fermented LLV beverage had a higher sourness than the original beverage. In general, no significant differences were found between beverages derived from the same raw material but fermented using different bacteria strains.

Volatile composition results also suggested that the main differences among samples were due to the beverage type. However, fermentation had a significant role in altering the composition of both dairy milk and rice beverage samples. Table 4 shows the volatile compounds, grouped by chemical families, found in the different samples. Dairy milk and its fermented samples were characterized by having a volatile profile in which different ketones, acids, esters, hydrocarbons, and lactones were present. However, the concentration of total acids (e.g., cheese, fatty, sour) significantly increased during fermentation. These results, as well as the sensory data, corresponded with the volatile composition previously found in milk and yogurt, characterized by the presence of δ-decalactone and different organic acids, among others [37,38]. The concentration of specific volatile compounds such as dimethyl sulfone, octanoic and decanoic acid ethyl esters (apricot, floral, grape, oily, pear), δ-decalactone (musty, fatty, fruity), or limonene (lemon, orange, citrus, sweet) significantly increased in the LLV fermented sample, suggesting a deeper transformation of the raw material due to the microbial activity of this bacterial combination. On the other hand, some ketones, such as 2-nonanone, increased in the CH dairy milk fermented sample, providing cheese, coconut, or oily aromas to this beverage. Rice beverage was characterized by a high concentration of aldehydes (e.g., fatty, green, oily, fruity, apple), confirming a previous study in which this chemical family was reported as being predominant in different commercial rice beverages [14]. Fermentation was able to significantly reduce the concentration of aldehydes, ketones, and alcohols in this matrix, as well as increasing the acid content. In general, total volatile compounds increased during fermentation in the dairy milk sample. At the same time, they decreased in the rice beverage, suggesting a completely different role of the process in both matrices. Previous research has reported a decrease in some volatile compounds responsible for off-flavors in plant-based beverages; for example, the levels of hexanal (beany flavor) were reduced with fermentation in peanut beverages [39]. Volatile compounds such as n-hexanal and n-hexanol, originating from the oxidation of plant lipids and potentially responsible for the beany/legume off-flavor of some plant-based beverages [14], were significantly reduced during the rice beverage fermentative process. Finally, confirming the microbiological and previous physicochemical findings, the LLV combination appeared to have a more pronounced influence on the transformation of both dairy and rice matrices. However, it exhibited a variable effect on the volatile compound profiles, either increasing or diminishing their presence.

**Figure 2:**
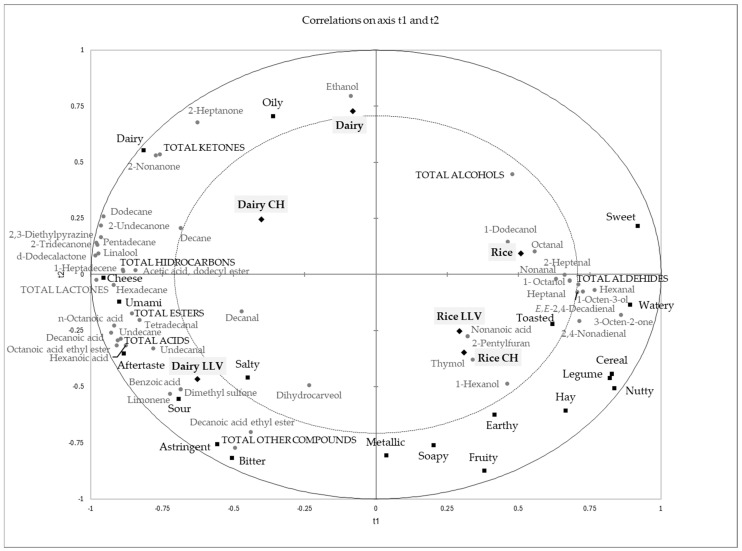
PLS regression map showing the relationship between volatile composition data correlated with sensory aromas that were significantly different among studied samples. R^2^Y = 0.753 and R^2^X = 0.687 in the first two components of the model.

Figure 2 shows the partial least square regression map explaining the relationship between volatile composition correlated with the sensory aromas that were significantly different among the studied samples; approximately 75% of the variation of the aroma sensory data was explained by the 69% variation of the volatile composition data. The first component clearly showed the differences due to the raw material, while the second component was linked to the fermentation process. The higher volatile complexity of the dairy samples, with higher total lactones, hydrocarbons, esters, and ketones, was associated with more intense dairy, cheese, and umami flavors. In contrast, the higher concentration of alcohols and aldehydes was related to the cereal, legume, and nutty notes of the rice-based samples. Although made from cereal, rice beverages had been previously reported as having leguminous notes, probably due to the presence of specific volatile compounds such as decanal [14].

Rising concerns about the environmental impact of dairy farming, ethical considerations, and medical needs such as lactose intolerance and cows’ milk protein allergy are driving consumers towards plant-based dairy alternatives [41]. However, the current market weight of plant-based alternatives is estimated at USD 2.2 billion, which only represents a small percentage of the global milk market, estimated at USD 1.7 trillion. One of the main reasons for this small market share may be the low acceptability of current plant-based alternatives [42]. Differences in the nutritional profiles of the different matrices (e.g., shown in Table 1) can influence the organoleptic profile of the product and its derivatives. Fermentation has been proposed as an effective strategy to develop new products and diminish the drawbacks of plant-based derivatives [15,26,32]. In the present study, to gain knowledge for developing plant-based products with desirable sensory attributes, the performance of typical commercial yogurt cultures (CH) and selected LAB strains (LLV) were compared in dairy milk and a rice beverage. Other plant-based beverages should be explored to confirm the capability of different bacteria strains to transform the raw materials. In addition, consumer studies should be conducted to determine the expectations and requirements of consumers on plant-based fermented products. Consideration should be given not only to the flavor characteristics of the products but also to appearance and other extrinsic factors that may influence consumer food choices.

## 4. Conclusions

Consumers’ increasing interest in plant-based yogurts and dairy alternatives faces a challenge: replicating the desired textures and flavors. This study aimed to explore this challenge by investigating the impact of the raw material and the fermentation starter on the final characteristics of fermented beverages. The results of the analyses showed the clear effect of the raw material on the aromatic profile of the final products (determined using GC–MS and sensory techniques). While confirming the matrix’s significant influence, the results of the research also suggested the need to properly select microbial starter combinations to drive the design of potentially probiotic drinks with diverse physicochemical and organoleptic properties. Further characterization of the selected strains holds the key to unlocking fermentation’s full potential in mimicking the nutritional, sensory, and textural qualities of dairy products.

## Figures and Tables

**Table 1:** Nutritional composition of samples to be fermented.

Nutritional Value (per 100 mL)	Dairy	Rice
Energy (kJ/kcal)	261/62	221/52
Total fats (g)	3.6	1
Saturated (g)	2.4	0.1
Total carbohydrate (g)	4.5	10
Sugars (g)	4.5	4
Protein (g)	3	0.3
Sodium (g)	0.13	0.07
Calcium (mg)	110	-

**Table 2:** Physicochemical characteristics (pH, TA, and TSS) after fermentation of each sample. Different lowercase letters within the rows indicate different post hoc groupings by Tukey’s HSD (*p* < 0.001); uppercase letters within the column indicate different post hoc groupings by Tukey’s HSD (*p* < 0.001).

Nutritional Value (per 100 mL)	Control	CH	LLV
pH			
Dairy	6.67 ± 0.05 (a A)	4.89 ± 0.08 (b A)	4.63 ± 0.02 (c A)
Rice	6.44 ± 0.04 (a B)	4.27 ± 0.12 (b B)	3.69 ± 0.07 (c B)
TA (g lactic acid/100 mL)			
Dairy	0.09 ± 0.01 (a A)	0.60 ± 0.02 (b A)	0.71 ± 0.01 (c A)
Rice	0.01 ± 0.01 (a B)	0.04 ± 0.01 (b B)	0.09 ± 0.01 (c B)
TSS (°Brix)			
Dairy	13.70 ± 0.36 (a A)	7.53 ± 0.14 (b B)	6.38 ± 0.28 (c B)
Rice	12.70 ± 0.13 (a B)	12.18 ± 0.17 (ab A)	11.88 ± 0.63 (b A)

**Table 3:** Results of the RATA test. Different letters within the rows indicate different post hoc groupings by Tukey’s HSD (*p* < 0.05).

Attribute	Dairy	Dairy LLV	Dairy CH	Rice	Rice LLV	Rice CH	*p*-Value
Sweet	3.8 (ab)	2.1 (c)	2.4 (bc)	4.9 (a)	3.4 (abc)	4.2 (a)	<0.0001
Salty	1.5	2.4	2.8	1.8	2.5	2.0	0.273
Bitter	1.4	2.4	2.1	1.6	2.0	2.1	0.307
Sour	2.0 (b)	4.9 (a)	4.0 (a)	1.9 (b)	4.0 (a)	2.5 (b)	<0.0001
Umami	2.1 (ab)	3.4 (a)	3.3 (ab)	2.1 (ab)	1.9 (b)	1.9 (b)	0.003
Cereal	1.4 (b)	2.3 (b)	1.7 (b)	5.3 (a)	4.1 (a)	3.9 (a)	<0.0001
Legume	1.1 (b)	1.4 (b)	1.6 (b)	3.1 (a)	2.5 (ab)	3.4 (a)	<0.0001
Nutty	1.5 (b)	1.8 (ab)	1.5 (b)	2.7 (a)	2.4 (a)	2.5 (a)	0.043
Earthy	1.2 (b)	1.8 (ab)	1.4 (ab)	2.0 (ab)	1.5 (ab)	2.7 (a)	0.026
Dairy	5.4 (a)	4.6 (a)	4.8 (a)	2.4 (b)	2.7 (b)	2.2 (b)	<0.0001
Fruity	1.4 (b)	2.6 (ab)	1.9 (ab)	2.6 (ab)	3.0 (a)	2.6 (ab)	0.017
Cheese-like	2.6 (bc)	4.1 (a)	3.9 (ab)	1.6 (c)	2.4 (c)	1.5 (c)	<0.0001
Hay-like	1.4	1.6	1.5	1.9	1.9	2.4	0.180
Toasted	1.4	1.4	1.6	2.1	1.4	2.3	0.165
Metallic	1.4	2.4	2.4	1.8	2.9	2.8	0.114
Soapy	1.2	1.9	2.1	1.8	2.4	2.3	0.331
Oily	2.1	1.9	2.3	2.1	1.7	1.7	0.871
Astringent	1.4 (b)	3.8 (a)	2.6 (ab)	2.2 (ab)	2.6 (ab)	2.2 (ab)	0.008
Graininess	1.7 (a)	2.4 (a)	2.3 (a)	1.3 (ab)	1.1 (b)	1.3 (ab)	0.020
Watery	3.9 (ab)	2.6 (bc)	1.7 (c)	4.7 (a)	5.1 (a)	4.6 (a)	<0.0001
Thickness	2.4 (b)	4.4 (a)	5.1 (a)	1.4 (b)	1.7 (b)	1.9 (b)	<0.0001
Lumpiness	1.5 (ab)	2.1 (a)	2.1 (a)	1.0 (b)	1.1 (b)	1.4 (ab)	0.031
Aftertaste	3.5 (ab)	4.7 (a)	4.5 (ab)	3.4 (b)	3.8 (ab)	3.6 (ab)	0.007

**Table 4:** Volatile composition of the raw and fermented samples (mg/kg). Different letters within the rows indicate different post hoc groupings by Tukey’s HSD (*p* < 0.05).

Compound	RI (exp)	RI (lit)	Dairy	Dairy LLV	Dairy CH	Rice	Rice LLV	Rice CH	*p*-Value	Descriptor
1-Hexanol	857	870	n.d. (b)	n.d. (b)	n.d. (b)	n.d. (b)	0.37 (a)	0.99 (a)	0.038	green; herbaceous; woody; sweet
1-Heptanol	958	970	n.d.	n.d.	n.d.	0.17	0.24	0.08	0.073	apple; apricot; coconut; musty; oily; woody
1-Octen-3-ol	970	978	n.d. (c)	n.d. (c)	n.d. (c)	0.28 (a)	0.15 (c)	0.12 (b)	<0.0001	cheese; creamy; earthy; herbaceous; vegetable
1-Octanol	1059	1075	n.d. (b)	n.d. (b)	n.d. (b)	0.49 (a)	n.d. (b)	0.06 (b)	<0.0001	orange; floral
1-Dodecanol *	1512	1478	0.86 (ab)	0.69 (b)	0.68 (ab)	0.84 (ab)	0.15 (b)	0.17 (a)	0.015	coconut; honey; fatty; earthy; soapy; waxy
Total Alcohols			0.86 (b)	0.69 (b)	0.68 (b)	1.47 (ab)	0.77 (b)	2.98 (a)	0.005	
Hexanal	795	785	n.d. (b)	n.d. (b)	n.d. (b)	0.04 (a)	0.12 (b)	0.69 (b)	0.001	fatty; green
Heptanal	893	896	n.d. (c)	n.d. (c)	n.d. (c)	0.40 (a)	n.d. (c)	0.14 (b)	<0.0001	oily; fruity; woody; fatty; nutty
2-Heptenal	944	954	n.d. (b)	n.d. (b)	n.d. (b)	0.48 (a)	n.d. (b)	0.13 (b)	<0.0001	apple; lemon; green; fatty; spicy; vegetable
Octanal	993	1007	n.d. (b)	n.d. (b)	n.d. (b)	0.66 (a)	n.d. (b)	n.d. (b)	<0.0001	honey; fatty; citrus
Nonanal	1093	1102	0.14 (bc)	0.16 (bc)	0.17 (bc)	1.15 (a)	0.09 (c)	0.48 (b)	<0.0001	apple; coconut; grape; grapefruit; lemon; lime;
Decanal	1193	1207	0.06 (ab)	0.11 (a)	0.10 (ab)	0.09 (ab)	0.02 (b)	0.08 (ab)	0.024	floral; citrus; sweet
2,4-Nonadienal	1201	1215	n.d. (b)	n.d. (b)	n.d. (b)	0.08 (a)	n.d. (b)	0.08 (a)	<0.0001	melon; fatty; floral; fishy; vegetable; meaty
*E*-2-Decenal	1253	1264	0.02	0.03	0.04	n.d.	n.d.	0.05	0.052	oily; orange; floral; citrus; fatty; waxy; green
Undecanal	1303	1310	n.d. (b)	0.02 (a)	0.03 (a)	n.d. (b)	n.d. (b)	0.01 (ab)	<0.001	orange; fatty; rose; waxy
*E,E*-2,4-Decadienal	1314	1314	n.d. (b)	n.d. (b)	n.d. (b)	0.14 (a)	n.d. (b)	0.07 (ab)	0.001	fatty; citrus; meaty
Dodecanal	1435	1420	0.04	0.07	0.07	0.03	0.08	0.06	0.187	herbaceous; waxy; floral; sweet
Tetradecanal	1597	1618	0.02 (ab)	0.04 (a)	0.03 (ab)	0.02 (ab)	0.01 (b)	0.03 (ab)	0.022	
Total Aldehydes			0.27 (b)	0.44 (b)	0.44 (b)	6.63 (a)	1.36 (b)	1.81 (b)	<0.0001	
2-Heptanone	879	875	0.49 (ab)	0.28 (bc)	0.68 (a)	0.22 (cd)	n.d. (d)	0.06 (d)	<0.0001	banana; cinnamon; spicy; fruity
Dimethyl sulfone	906	912	n.d. (b)	5.22 (a)	n.d. (b)	n.d. (b)	n.d. (b)	n.d. (b)	0.005	
3-Octen-2-one	1026	1037	n.d. (b)	n.d. (b)	n.d. (b)	0.31 (a)	0.14 (ab)	0.12 (ab)	0.003	berry; butter; lemon; nutty; herbaceous; vegetable
2-Nonanone	1078	1091	0.42 (b)	0.29 (b)	0.69 (a)	n.d. (c)	n.d. (c)	n.d. (c)	<0.0001	cheese; coconut; oily; herbaceous; floral; fruity
2-Undecanone	1287	1291	0.17 (b)	0.27 (a)	0.28 (a)	n.d. (c)	n.d. (c)	n.d. (c)	<0.0001	iris; citrus
2-Tridecanone *	1528	1498	0.06 (b)	0.10 (a)	0.09 (a)	n.d. (c)	n.d. (c)	n.d. (c)	<0.0001	spicy; herbaceous
Total Ketones			1.14 (b)	0.99 (bc)	1.73 (a)	0.52 (cd)	0.14 (d)	0.19 (d)	<0.0001	
Hexanoic acid	964	965	0.11 (b)	3.42 (a)	3.14 (a)	n.d. (b)	0.85 (b)	0.41 (b)	<0.0001	cheese; fatty; sour
Benzoic acid	1153	n.d.	n.d. (b)	0.31 (a)	n.d. (b)	n.d. (b)	n.d. (b)	n.d. (b)	<0.001	balsam
n-Octanoic acid	1160	1182	0.13 (b)	2.54 (a)	2.06 (a)	n.d. (b)	0.03 (b)	0.02 (b)	<0.0001	oily
Nonanoic acid	1262	1260	n.d. (b)	n.d. (b)	n.d. (b)	n.d. (b)	0.04 (a)	n.d. (b)	0.023	cheese; waxy
Decanoic acid	1378	1380	0.05 (c)	0.56 (a)	0.34 (b)	n.d. (c)	n.d. (c)	n.d. (c)	<0.0001	fatty; citrus
Total Acids			0.30 (b)	6.83 (a)	5.54 (a)	n.d. (b)	0.91 (b)	0.43 (b)	<0.0001	
Decane	992	1000	0.18 (a)	0.16 (a)	0.17 (a)	n.d. (b)	n.d. (b)	0.17 (a)	0.001	
Undecane	1090	1100	0.02 (b)	0.12 (a)	0.04 (ab)	n.d. (b)	n.d. (b)	n.d. (b)	0.002	
Dodecane	1189	1200	0.36 (a)	0.44 (a)	0.39 (a)	0.15 (b)	0.15 (b)	0.15 (b)	<0.001	
Tridecane	1297	1300	0.06	0.10	0.13	0.08	0.05	0.09	0.310	
1-Tetradecene	1414	1398	0.15	0.27	0.32	0.18	0.04	0.21	0.457	
Tetradecane *	1426	1400	0.18	0.41	0.46	0.36	0.18	0.30	0.321	
Pentadecane *	1534	1500	0.02 (ab)	0.04 (a)	0.03 (a)	n.d. (b)	n.d. (b)	n.d. (b)	0.001	
1-Heptadecene *	1588	n.d.	0.07 (ab)	0.11 (a)	0.08 (ab)	0.04 (ab)	0.01 (b)	0.05 (ab)	0.042	
Hexadecane	1592	1600	0.05 (ab)	0.08 (a)	0.07 (ab)	0.03 (ab)	0.02 (b)	0.04 (ab)	0.024	
Total Hydrocarbons			1.09 (ab)	1.74 (a)	1.69 (a)	0.84 (b)	0.45 (b)	0.99 (ab)	0.048	
Octanoic acid ethyl ester	1185	1197	0.02 (bc)	0.08 (a)	0.04 (b)	n.d. (c)	n.d. (c)	0.02 (bc)	<0.0001	apricot; floral; pear; pineapple
Decanoic acid ethyl ester	1417	1399	n.d. (b)	0.05 (a)	n.d. (b)	n.d. (b)	n.d. (b)	0.03 (ab)	0.006	grape; oily; pear
Dodecanoic acid, methyl ester *	1549	1521	0.16	0.24	0.23	0.12	0.04	0.15	0.106	coconut; creamy; soapy; wax
Acetic acid, dodecyl ester	1595	1606	0.02 (ab)	0.02 (a)	0.02 (ab)	0.01 (ab)	0.01 (b)	0.01 (ab)	0.040	
Total Esters			0.19 (ab)	0.40 (a)	0.28 (ab)	0.13 (ab)	0.05 (b)	0.21 (ab)	0.024	
δ-Octalactone	1270	1288	0.03	0.23	0.16	n.d.	n.d.	n.d.	0.366	coconut; creamy; herbaceous; peach
δ-Decalactone	1521	1524	0.20 (b)	0.40 (a)	0.32 (ab)	n.d. (c)	n.d. (c)	n.d. (c)	<0.0001	musty; fatty; fruity
Total Lactones			0.23 (bc)	0.63 (a)	0.48 (ab)	n.d. (c)	n.d. (c)	n.d. (c)	<0.0001	
2-Pentylfuran	979	993	n.d. (b)	n.d. (b)	n.d. (b)	n.d. (b)	0.73 (a)	n.d. (b)	0.015	green; vegetable
*p*-Cymene	1013	1020	n.d.	n.d.	n.d.	n.d.	n.d.	0.21	0.458	citrus
Limonene	1018	1026	0.47 (b)	1.59 (a)	0.61 (b)	0.50 (b)	0.60 (b)	0.48 (b)	<0.001	lemon; orange; citrus; sweet
2,3-Diethylpyrazine *	1065	n.d.	0.03 (b)	0.05 (a)	0.03 (ab)	n.d. (c)	n.d. (c)	n.d. (c)	<0.0001	hazelnut; meaty; earthy
Terpinen-4-ol	1165	1177	n.d.	n.d.	n.d.	n.d.	n.d.	0.03	0.458	grapefruit; lemon; pepper; herbaceous; spicy
Linalool	1088	1099	0.04 (b)	0.07 (a)	0.04 (ab)	n.d. (c)	n.d. (c)	n.d. (c)	<0.0001	lemon; orange; floral; citrus; sweet
Dihydrocarveol	1181	1190	n.d. (c)	0.09 (b)	0.09 (b)	n.d. (c)	n.d. (c)	0.18 (a)	<0.0001	
Thymol	1291	1292	n.d. (b)	n.d. (b)	n.d. (b)	n.d. (b)	n.d. (b)	0.03 (a)	0.024	woody; fruity; sweet; minty; earthy; spicy
Total Other Compounds			0.54 (b)	1.80 (a)	0.79 (ab)	0.50 (b)	1.33 (ab)	0.93 (ab)	0.011	
Total Volatiles			4.61 (b)	12.90 (a)	11.61 (ab)	10.09 (ab)	5.00 (b)	7.54 (ab)	0.009	

Legend: Concentrations of aroma compounds were calculated by the application of the internal standard method and expressed as mg kg^−1^ resulting from the relative peak area to the internal standard (2-methyl-3-heptanone, 0.33 mg kg^−1^). Abbreviation n.d. to mean “not determined”. Volatile compounds were identified using three methods: GC–MS retention time of the chemical pure compounds, retention indexes (RI) calculated using a commercial alkane standard mixture (Literature RI from NIST [17]), and comparison of the compound mass spectrum with those of NIST database. Asterisks mark those compounds that were tentatively identified using only two of these three methods. Descriptors from SAFC [40].

## Data Availability

The original contributions presented in the study are included in the article, further inquiries can be directed to the corresponding author.
